# Acid-Tolerant Moderately Thermophilic Methanotrophs of the Class *Gammaproteobacteria* Isolated From Tropical Topsoil with Methane Seeps

**DOI:** 10.3389/fmicb.2016.00851

**Published:** 2016-06-15

**Authors:** Tajul Islam, Vigdis Torsvik, Øivind Larsen, Levente Bodrossy, Lise Øvreås, Nils-Kåre Birkeland

**Affiliations:** ^1^Department of Biology, University of BergenBergen, Norway; ^2^Uni Environment, Uni ResearchBergen, Norway; ^3^Oceans and Atmosphere, CSIRO, HobartTAS, Australia; ^4^Centre for Geobiology, University of BergenBergen, Norway

**Keywords:** terrestrial, *Gammaproteobacteria*, methanotroph, acid-tolerant, moderately thermophilic, pMMO

## Abstract

Terrestrial tropical methane seep habitats are important ecosystems in the methane cycle. Methane oxidizing bacteria play a key role in these ecosystems as they reduce methane emissions to the atmosphere. Here, we describe the isolation and initial characterization of two novel moderately thermophilic and acid-tolerant obligate methanotrophs, assigned BFH1 and BFH2 recovered from a tropical methane seep topsoil habitat. The new isolates were strictly aerobic, non-motile, coccus-shaped and utilized methane and methanol as sole carbon and energy source. Isolates grew at pH range 4.2–7.5 (optimal 5.5–6.0) and at a temperature range of 30–60°C (optimal 51–55°C). 16S rRNA gene phylogeny placed them in a well-separated branch forming a cluster together with the genus *Methylocaldum* as the closest relatives (93.1–94.1% sequence similarity). The genes *pmoA, mxaF*, and *cbbL* were detected, but *mmoX* was absent. Strains BFH1 and BFH2 are, to our knowledge, the first isolated acid-tolerant moderately thermophilic methane oxidizers of the class *Gammaproteobacteria*. Each strain probably denotes a novel species and they most likely represent a novel genus within the family *Methylococcaceae* of type I methanotrophs. Furthermore, the isolates increase our knowledge of acid-tolerant aerobic methanotrophs and signify a previously unrecognized biological methane sink in tropical ecosystems.

## Introduction

Microorganisms in tropical ecosystems play a crucial role for biogeochemical cycling as well as controlling terrestrial greenhouse gas fluxes, and have therefore impact on global climate regulation. Methane is a strong greenhouse gas and the major biogenic source, which is the end product of microbial degradation of organic matter in anoxic environments. Major sources of abiogenic methane are underground reservoirs in geothermal regions, where methane is released to the atmosphere through seeps, gas venting, and degassing of spring water (Etiope and Klusman, 2002; Aronson et al., 2013; Nazaries et al., 2013). In some regions, like the gas fields in Northeast Bangladesh, release of natural methane from tropical soils are caused by previous surface gas blowouts (Khan and Nasir, 2014). Such methane seeps influence the structure and function of microbial communities, and have importance for the global carbon cycle. Methane-oxidizing bacteria (MOB) or methanotrophs serve as a methane sink that suppress methane emissions to the atmosphere from various ecosystems, and contribute extensively to the global methane budget. Methanotrophic community structure and activity have been studied in terrestrial tropical habitats such as rice paddy fields (Alam and Jia, 2012; Dianou et al., 2012), peat soils (Arai et al., 2014), and upland soils (Knief et al., 2005) as well as in tropical shallow methane seep sediments (Wasmund et al., 2009). These studies, which have applied analyses of methane fluxes and populations of methanotrophs, support evidences for diverse communities of aerobic methanotrophs and the existence of novel uncultured MOBs. Although, several methanotrophs from tropical ecosystems have been isolated (Geymonat et al., 2011; Islam et al., 2015; Khalifa et al., 2015), but knowledge about methanotrophs and their responses to environmental factors in such ecosystems is still limited (Aronson et al., 2013). Furthermore, no previous studies have reported on methanotrophic communities in high temperature tropical soils (around 50°C) with high methane concentrations due to surface gas blowouts. Such investigations are essential to understand the microbial impact and the effect of environmental factors on methane fluxes. In particular, studies of isolated methanotrophs from such tropical ecosystems will expand our knowledge about the genetics, biochemistry, and ecophysiology of this functional group.

The methane oxidizing microbes constitute a unique group defined by their ability to utilize methane as sole source for carbon and energy, and they are isolated from a wide variety of habitats (Hanson and Hanson, 1996). Several clusters of uncultivated methane oxidizers have been detected in both tropical and temperate ecosystems, indicating the need for further cultivation efforts to obtain new pure cultures of methanotrophs (Knief, 2015). Until now, twenty two genera of proteobacterial methanotrophs (Deutzmann et al., 2014; Hoefman et al., 2014; Khalifa et al., 2015; Tavormina et al., 2015) and two genera of verrucomicrobial extreme acidophilic methanotrophs (Op den Camp et al., 2009; Sharp et al., 2014b; van Teeseling et al., 2014) are validly described. In the phylum *Proteobacteria*, MOB are assigned to four families *Methylothermaceae, Methylococcaceae, Methylocystaceae* and *Beijerinckiaceae*, which are described based on phylogeny, chemotaxonomy, arrangement of intracytoplasmic membrane (ICM), DNA mol% G+C, and pathways for carbon assimilation (Hanson and Hanson, 1996; Trotsenko and Murrell, 2008; Hirayama et al., 2014).

Commonly used molecular markers for MOB are the key functional marker genes *pmoA* (the particulate methane monooxygenase), *mmoX* (the soluble methane monooxygenase), and *mxaF* (the methanol dehydrogenase). In particular, the *pmoA* gene, encoding the 27 kDa polypeptide of the particulate methane monooxygenase, has been used as a most frequently phylogenetic marker for identifying aerobic methanotrophs except those in the family *Beijerinckiaceae*. The *mxaF* gene was also suggested as a functional and phylogenetic marker for proteobacterial methanotrophs and methylotrophs in natural environments (McDonald and Murrell, 1997; McDonald et al., 2008; Lau et al., 2013). Another functional gene, *cbbL*, encodes the large subunit of the ribulose-1,5-bisphosphate carboxylase/oxygenase (RuBisCO), which is a key enzyme responsible for autotrophic CO_2_ fixation of the Calvin-Benson-Bossham (CBB) cycle. The *cbbL* gene has been frequently applied for analyzing marine and hypersaline microbial communities (Tourova et al., 2010) as well as for identification of methanotrophic *Methylococcus*-like strains and *Methylocaldum* species (Baxter et al., 2002).

*Methylococcus capsulatus* was the first described heat-tolerant methanotroph within the family *Methylococcaceae*, growing at temperatures up to 50°C and at pH range 5.5–7.5 (Foster and Davis, 1966; Whittenbury et al., 1970). Several moderately thermophilic methanotrophs of the class *Gammaproteobacteria*, such as *Methylocaldum szegediense, Methylocaldum tepidum, Methylocaldum gracile, Methylocaldum* sp. strain H-11, *Methylocaldum* sp. strain O-12, *Methylocaldum marinum* and *Methylothermus thermalis* (Bodrossy et al., 1997; Eshinimaev et al., 2004; Tsubota et al., 2005; Takeuchi et al., 2013) were subsequently isolated from thermal environments and marine sediments. In addition, a true thermophilic methanotroph named strain HB, which grew on methane up to 72°C, was isolated from underground hot springs in Hungary (Bodrossy et al., 1999). Sequence comparison indicated that the *pmoA* gene was most closely related to known *Methylococcus* and *Methylocaldum* species. Unfortunately, this strain is no longer available (Tsubota et al., 2005). Moreover, using stable-isotope probing in combination with 16S rRNA gene pyrotag sequencing, *Methylocaldum* species were identified in sediments of warm geothermal springs ranging in temperature from 22 to 45°C (Sharp et al., 2014a). Recently, three mesophilic *Methylocaldum*-like methanotrophs (growth range 8–35°C) of the family *Methylococcaceae* were isolated from different geographic regions, and these isolates fell into a cluster consisting of the genera *Methylocaldum-Methylococcus-Methyloparacoccus-Methylogaea* (Islam et al., 2015).

Extreme acidophilic methanotrophs of the phylum *Verrucomicrobia* have been discovered by cultivation-dependent and -independent approaches (Sharp et al., 2014b; van Teeseling et al., 2014). Three thermoacidophilic verrucomicrobial methanotrophs (pH growth range 0.5–6.0 and optimum temperature 55–60°C) have been described, thus extending the phylogeny of true thermoacidophilic methanotrophs beyond the phylum *Proteobacteria*. The verrucomicrobial methanotrophs belong to the family *Methylacidiphilaceae*, and were obtained from extreme geothermal environments at different geographic regions. (Dunfield et al., 2007; Pol et al., 2007; Islam et al., 2008; Op den Camp et al., 2009). Mesophilic verrucomicrobial methane oxidizers have also been characterized, which shows that methanotrophs in this phylum are more diverse and widespread than earlier anticipated (van Teeseling et al., 2014). Slightly acidophilic methanotrophs belonging to the genera *Methylocella, Methylocapsa*, and *Methyloferula* of the class *Alphaproteobacteria* are abundant in peat bog ecosystems and able to grow between pH 4.2 and 7.5 (Dedysh et al., 2007; Dedysh, 2009; Vorobev et al., 2011). In addition, two strains of acid-tolerant or acidophilic type I methanotrophs, *Methylomonas* strain M5 and strain M200 of the family *Methylococcaceae* were isolated from peat ecosystems, and they are able to grow below pH 4.5 (Kip et al., 2011). Recently, an acid-tolerant type I methanotroph, *Methylomonas paludis* strain MG30, was also isolated from an acidic *Sphagnum* peat bog in north-eastern Russia and had a pH growth range of 3.8–7.3 (Danilova et al., 2013).

Hitherto, no true thermoacidophilic or acid-tolerant moderately thermophilic gammaproteobacterial methanotrophs have been described. The present study describes the recovery and initial characterization of two novel methane oxidizers belonging to the class *Gammaproteobacteria* isolated from a methane-rich tropical topsoil.

## Materials and Methods

### Sampling

**FIGURE 1:**
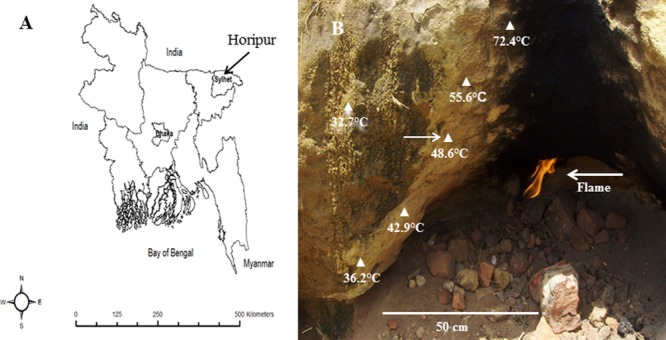
**Sampling site. (A)** Map of Bangladesh showing the sampling place. **(B)** A close-up photograph (with temperature gradient) of terrestrial tropical topsoil by leakage natural methane gas at Horipur, Sylhet, north-east Bangladesh. The arrow indicates the sampling site.

Sampling was performed in June 2007, and again in November 2009. Tropical topsoil (5 cm depth) was collected from the side of a small cave, which was heated by a flame of natural methane gas leakage or surface gas blowout. The place is called Horipur (village: Utlarpar) and is situated about 20 km from Sylhet in northeast Bangladesh (24° 98′07″ N, 92° 03′29″ E; **Figure 1**). The *in situ* soil temperature was measured by a digital temperature sensor (Digitron, 2000T, Sifam Instruments, UK). The pH of topsoil (1:1, soil:water) was measured by a pH meter (MP220, Mettler Toledo). Soils were placed in Falcon tubes and frozen at -20°C on the sampling day.

### Isolation and Cultivation

To enrich for moderately thermophilic MOB, low-salt mineral medium supplemented with NH_4_Cl (0.1 g L^-1^; LMA) was used. This medium is 10 times more diluted than the AMS medium (Whittenbury et al., 1970). No vitamins were included in the medium. The pH was adjusted to 6.0 and 6.5 with 1 M HCl or 1 M NaOH. In addition, low-salt mineral medium with NH_4_Cl replaced by KNO_3_ (0.1 g L^-1^) was applied for methanotrophic enrichments. Three gram of soil was added to 20 mL medium in 120 mL sterile serum bottles. The bottles were closed with a butyl rubber cap with an aluminum crimp seal. A mixture of methane (purity 99.5%, Yara Praxair, Oslo, Norway) and air was added aseptically through a syringe to achieve 80 and 20% concentration of methane and air, respectively, in the headspace. The flasks were shaken at 125 rpm on a rotary shaker kept at 50°C. After 1 week incubation, the medium became visibly turbid and growth of the enrichment cultures was verified by phase-contrast microscopy (Eclipse E400 microscope, Nikon Corporation, Tokyo, Japan). Then, 1 mL of the cultures were transferred to fresh LMA and incubated at the same conditions. After five passages of the enrichments, the cultures were serial diluted (10^-5^ to 10^-8^), and 0.1 mL aliquots of each dilution were spread onto plates containing a mixture of LMA and gelrite (20 g L^-1^; Gelzan^TM^ CM, Sigma-Aldrich, Corporation, St. Louis, MO, USA) or agar (Difco). The plates were incubated at 50°C in jars filled with a methane/air (4:1) gas mixture. Individual colonies were picked and re-streaked onto fresh plates and re-incubated. Finally, single colonies were transferred to fresh liquid LMA and incubated for 1 week with methane and air.

### Purity Verification of Isolates and Electron Microscopy

The purity of isolates was examined by phase-contrast microscopy, and further verified by formation of uniform colonies on LMA gelrite plates and absence of growth of heterotrophs in LMA supplemented with Luria-Bertani broth (1–5% v/v), glucose (10 mM), acetate (18 mM), pyruvate (10 mM), succinate (10 mM), ethanol (17 mM), yeast extract (0.1%), and by streaking onto R2A (Reasoner’s 2A) agar plates (van der Linde et al., 1999). The isolation process was monitored by polymerase chain reaction (PCR) amplification and sequencing of the partial 16S rRNA gene until each of the isolates gave a clean and reproducible sequence read. Exponentially growing cultures of strain BFH1 were collected by centrifugation and applied for transmission electron microscopy analysis (a Jeol-1230 electron microscope, at 60 KV, Tokyo, Japan), as described previously (Islam et al., 2015).

### Utilization of Carbon and Nitrogen Sources

Capability to utilize various organic compounds was tested in liquid LMA medium supplemented with the following autoclaved or filter-sterilized substrates at 10 mM concentration: urea, acetate, glucose, pyruvate, lactate, malate, ethanol, succinate, sucrose, fructose, maltose, mannitol, and sorbitol. Growth on the C1 substrates methanol, formate, methylamine, and formaldehyde were tested at concentrations from 0.03 to 0.2% (v/v). Luria-Bertani broth and yeast extract were also tested as supplements to LMA. During incubation, bottles were capped with butyl-rubber stoppers to prevent vaporization. The growth of the strains was also tested with nitrogen-free LMA (without NH_4_Cl or KNO_3_) in triplicate 120-mL serum bottles where N_2_ from the air (20% air in the headspace) was the only N-source. The bottles were incubated for 2 weeks.

### Optimum pH, Temperatures, and Salt Concentrations

The temperature range and the optimum temperature for growth were tested at 20, 25, 28, 30, 35, 37, 40, 45, 48, 50, 54, 58, 60, 62, 65, and 70°C (growth at pH 6.0). In order to evaluate the effects of pH we measured the growth rates of the isolates at the optimum temperature 51°C by counting cells in the phase-contrast microscope. Growth rates were assessed at the following pH values: 3.0, 3.5, 4.0, 4.2, 4.3, 4.5, 5.0, 5.5, 6.0, 6.5, 6.8, 7.2, 7.5, 7.8, 8.0, 8.5, 9.0. To determine the salt-dependence of growth, NaCl (0.1, 0.5, 1.0, 1.5, 2.0, and 3.0%, w/v) was added to the medium. The generation time was calculated during exponential growth phase. The optical cell density (at 600 nm) was measured at optimum temperature (51°C) and at pH 6.0. Antibiotic sensitivity was tested and the following antibiotics (μg mL^-1^) were used: ampicillin, 10; tetracycline, 10; kanamycin, 30; streptomycin, 10; erythromycin, 10; and nalidixic acid, 30. Growth was evaluated after 10 days incubation.

### Acetylene Inhibition Test, Naphthalene Assay, Fatty Acid Profiles, and DNA mol% G+C

The naphthalene-oxidation assay, testing for the presence of soluble form of methane monooxygenase (sMMO), was performed with a liquid LMA culture without copper (Graham et al., 1992). *M. capsulatus* strain Bath was used as positive control of the assay. The effects of acetylene were examined by adding 4% (vol/vol) acetylene in the headspace of three replicate flasks containing cultures of the isolates in early exponential phase growing in LMA. To verify the acetylene inhibition test, *M. capsulatus* strain Bath and *Methylacidiphilum kamchatkense* strain Kam1 were used as positive controls (Islam et al., 2008). Phospholipid fatty acid (PFLA) and DNA mol% G+C analyses were performed at the DSMZ (Deutsche Sammlung von Mikroorganismen und Zellkulturen GmbH, Braunschweig, Germany).

### PCR Amplification and Southern Blot Hybridization

Genomic DNA was extracted with GenElute bacterial genomic DNA kit (Sigma-Aldrich) and used as template for PCR amplification of the 16S rRNA gene using the universal bacterial primers 27f and 1492r (Weisburg et al., 1991). The functional genes *pmoA, mmoX, mxaF*, and *cbbL* were amplified using primers listed in Supplementary Table S1. PCR amplifications were carried out in 50 μL volumes in a Veriti 96 well Thermal Cycler (Applied Biosystems, Carlsbad, CA, USA) using Dynzyme^TM^ High-Fidelity DNA Polymerase (Finnzymes, Finland). All PCR reactions were performed as described previously (Islam et al., 2015). Genomic DNA was also used for Southern blot analysis and DNA from *M. capsulatus* strain Bath, and *M. kamchatkense* strain Kam1 were applied as positive and negative controls, respectively. Genomic DNA was digested with EcoRI and HindIII and separated by agarose gel electrophoresis and blotted onto Hybond-N nylon membranes (Amersham Biosciences, Piscataway, NJ, USA). Hybridization probes were prepared by PCR using the *pmoA* and *mmoX* primer sets (Supplementary Table S1) and labeled with [α-^32^P]dCTP using DNA label kit (Amersham Biosciences) as previously described (Baxter et al., 2002; Islam et al., 2008).

### Phylogenetic Analysis

The nucleotide sequences of the functional genes were translated into amino acid sequences using the ExPASy Translate tool^1^. 16S rRNA sequences and the deduced protein sequences (PmoA, MxaF, and CbbL) were compared with available sequences in the GenBank database using BLASTn and BLASTp (the NCBI tools), respectively. Phylogenetic analyses based on 16S rRNA and PmoA sequences were carried out by aligning sequences using CLUSTAL W algorithm as implemented in MEGA6 software package (Tamura et al., 2013). Phylogenetic trees were constructed by different methods such as the Neighbor-Joining, Maximum-Likelihood, and Minimum-Evolution. Distances were determined using either Kimura 2-parameter models, Jones-Taylor/Thornton or Maximum composite likelihood method also implemented in MEGA6 software. The 16S rRNA, particulate methane monooxygenase (*pmoA*), methanol dehydrogenase (*mxaF*), and the ribulose-1,5-bisphosphate caboxylase/oxygenase (*cbbL*) gene sequences of strains BFH1 (GQ130271, GQ130270, GQ130269, KP878519) and BFH2 (KP828774, KT921321, KT921322, and KT932010) respectively, were deposited in the GenBank.

## Results

### Isolation of Acid-Tolerant Moderately Thermophilic MOB

The *in situ* temperature and pH at the sampling site were 48.6°C and 5.0, respectively. Very poor growth of enrichment cultures of methanotrophic bacteria was observed in a low-salt mineral medium supplemented with KNO_3_ whereas better growth was observed in LMA medium (with NH_4_Cl). The enrichment cultures in LMA medium reached a density of about 10^8^ cells mL^-1^ after a second transfer. Strains BFH1 and BFH2 were isolated after subculturing in fresh LMA five times and growth of single colonies on gelrite plates. Small white colonies of the strains BFH1 (about 0.8–1 mm in diameter) and BFH2 (about 1–1.4 mm in diameter) were observed on gelrite plates after 10 days incubation in methane:air (80:20) atmosphere. On agar plates, no colonies appeared even after 3 weeks of incubation. Growth with methane or methanol (0.05%) in liquid cultures first developed white turbidity and later became semitransparent. No growth of the strains was observed in the absence of methane or in the presence of methane under anaerobic conditions.

### Morphological Properties

**FIGURE 2:**
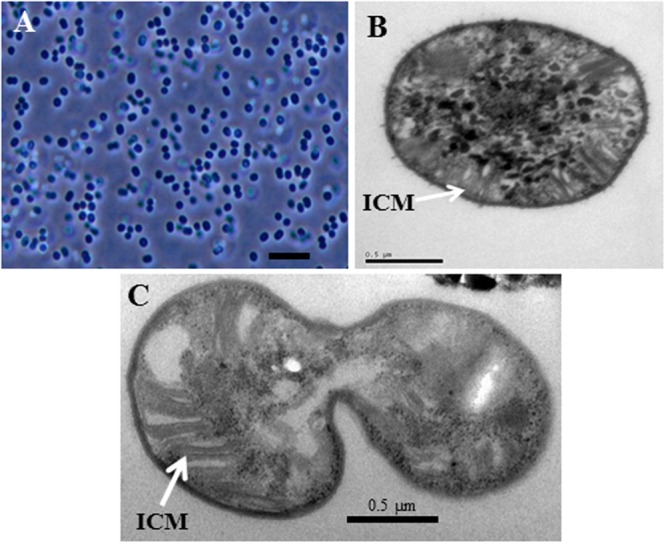
**Morphology of strain BFH1. (A)**, Phase-contrast photomicrograph of cells grown in LMA medium under methane at 51°C for 5 days. **(B,C)** Transmission electron micrograph of the strain BFH1. Ultrathin sections showing intracytoplasmic membrane arrangements (ICM). Bars, 3 μm **(A)**, 0.5 μm **(B,C)**.

Morphological characteristics of strains BFH1 and BFH2 as compared to thermotolerant, thermophilic, and acid-tolerant proteobacterial methanotrophs are presented in **Table 1**. Cells of the isolates (in exponential and early stationary phase) were non-motile and had slightly elongated coccus-shape, 1.0–2.0 μm in length and 1.0–1.3 μm in diameter. They usually occurred single or in pairs, and did not form chains or aggregates (**Figure 2A**, strain BFH1). No cyst-like cells were detected. Cells were Gram-negative and reproduced by binary fission. By transmission electron microscopy of BFH1, extensive ICM structures, a typical feature of pMMO-possessing proteobacterial methanotrophs, was confirmed (**Figures 2B,C**). No flagella were observed.

**Table 1:** Comparison of the major characteristics of the acid-tolerant moderately thermophilic strains BFH1 and BFH2 with themotolerant, thermophilic, and acid-tolerant methane oxidizing bacteria of the families *Methylococcaceae* and *Methylothermaceae*.

Characteristic	Strains BFH1 and BFH2	^1^*Methylocaldum* spp.	*^2^Methylothermus thermalis*	^3^*Methylococcus capsulatus*	*^4^Methylomagnum ishizawai*	^5^Acid-tolerant strain M200
Cell morphology	Coccoids	Rods-pleomorphic	Coccoids	Coccoids	Rods	Cocci
Acidophilic condition	Acid-tolerant	Neutrophilic	Neutrophilic	Neutrophilic	Neutrophilic	Acid-tolerant
ICM arrangement	Type I	Type I	Type I	Type I	Type I	Type I
Motility	-	-	-	-	-	+
Cyst formation	-	+	-	-	+	-
Pigmentation	White	Brown/cream	White	Yellow	White	Pink
Methane oxidation	pMMO	pMMO	pMMO	pMMO/sMMO	pMMO	pMMO/PxmA
*mmoX*	-	+^a^	-	+	+	-
*mxa*F	+	+	+	+	nr	nr
*cbbL*	+	+	+	+	nr	nr
N_2_-fixation	+	+	-	+	-	+
Temperature (optimal) °C	30-60 (51-55)	20-61 (42-55)	37-67 (57-59)	20-47 (42-45)	20-37 (31-33)	4-30
**Growth of pH (optimal)**	**4.2-7.5 (5.5-6.0)^b^**	**6.0-8.5 (7.0-7.2)**	**6.5-7.5 (6.8)**^c^	**5.5-7.5 (6.5)**	**5.5-9.0 (6.8-7.4)**	**4.1-7.0 (5.5)**
Growth on methanol (0.1%)	+	-	+	+	-	+
Vitamin required	-	-	-	-	-	-
G+C content (mol%)	62.7	58.5	62.5	62.5	64.1	52.2^d^
Isolation source (pH)	Tropical topsoil (pH 5.0)	Manure, silage (pH 6.0)	Hot spring (pH 6.0)	Hot spring (pH 6.0)	Rhizosphere soil (pH 6.8)	*Sphagnum* mosses (pH 3.8–4.3)

*Reference for strains: **^1^**Bodrossy et al., 1997 and Eshinimaev et al., 2004; **^2^**Tsubota et al., 2005; **^3^**Bowman et al., 1993; ^4^Khalifa et al., 2015; **^5^**Kip et al., 2011. +, positive results; –, negative results; nr, not reported; ICM, intracytoplasmic membrane. ^a^Only *Methylocaldum marinum* strain S8^T^ (Takeuchi et al., 2013) possessed *mmoX* (encodes soluble methane monooxygenase). ^b^Growth on LMA with methane or methanol of strain BFH2 was observed at pH range 4.5-7.5. **^c^***Methylothermus subterraneus* strain HTM55^T^ grew at pH 5.2–7.5 (Hirayama et al., 2011). ^d^16S rRNA, *pmoA*, and *pxmA* genes sequences were used for computing measurement of DNA G+C content (mol%).*

*Significance of bold values: to compare pH growth among methane oxidizers.*

### Physiological Characteristics and Fatty Acid Profile

Physiological characteristics of strains BFH1 and BFH2 are presented in **Table 1**. The isolates were unable to grow on substrates containing multi-carbon compounds or in complex media, and utilized only methane and methanol as carbon and energy source. They could utilize methanol at a wide range of concentrations (0.05-0.2%), with an optimum concentration between 0.1 and 0.15%. Vitamins were not required for growth. Cells used ammonium or nitrate as nitrogen sources. Better growth was observed with NH_4_Cl than with KNO_3_ or NaNO_3_ in LMA medium. Growth in nitrogen-free liquid medium was observed but it was not as good as with NH_4_Cl. The strains were able to grow at NaCl concentrations up to 0.5% (w/v). The growth temperature range of both strains was 30-60°C (no growth occurred at 25 or 65°C) and the optimum growth temperature was 51–55°C. Growth of strains BFH1 and BFH2 was observed at pH range 4.2–7.5 and 4.5–7.5, respectively, and no growth was observed at pH 4.0 or 7.8. Fastest growth was observed at pH between 5.5 and 6.0 for BFH1, and 5.7 and 6.2 for BFH2 (Supplementary Figure S1). The estimated generation times of strains BFH1 and BFH2 under optimum conditions were 21 h (specific growth rate, 0.033 h^-1^) and 19 h (specific growth rate, 0.036 h^-1^), respectively. All the tested antibiotics inhibited growth and the naphthalene oxidation assay was negative. Acetylene is often applied as inhibitor for the methane oxidation process and growth on methane was completely blocked after addition of 4% (vol/vol) acetylene to the headspace. The fatty acid compositions of strain BFH1 and other related thermophilic, thermotolerant, and acid-tolerant methanotrophs are presented in **Table 2**. The major fatty acids in the strain BFH1 were C16:0 (54.38%), C17:0*cyc* (26.33%) and C16:1*ω*7 (13.83%).

**Table 2:** Cellular PLFA profiles comparison of the strain BFH1 and other phylogenetically related methanotrophic genera or species.

Fatty acids	1	2	3	4	5
C13:1					0.27
iC14:0	0.59		1.24		
C14:0	0.78	1.97		0.8–6.2	15.8
C14:0 2-OH			0.33		
C15:	0.80	3.51	2.07	0–1.7	1.56
C15:1ω8					0.22
C15:1ω6	0.12		0.17		
iC16:0	0.26				
C16:1ω11					5.46
**C16:1ω7c**	**13.83**	**3.46**	**10.6–23.1**	**47.3**
C16:1ω6c				3.9–12.3	8.03
C16:1ω5c	0.37			3.2–9.0	
C16:1ω5t				1.8–6.0	
**C16:0**	**54.38**	**63.67**	**37.24**	**33.5–56.0**	**19.6**
C16:1		11.90			
C16:0 3-OH	0.28	0.64			1.78
9-o-Me-C16:0		4.62			
**C17:0*cyc***	**26.33**	**8.99**	**4.71**	**0–14.0**	
aC17:0		0.68			
C17:0	0.46	0.34	2.52		
C17:1		0.43	0.19		
C17:1ω6	0.62	0.26			
C17:1ω7c				0–1.9	
9-o-Me-C17:0		0.60			
11-0-Me-17:0		0.60			
C17:0	0.31				
iC18:0		0.26		0.6–1.8	
C18:0		0.17	1.74	0–2.1	
C18:1ω7c	0.71		0.35	0–6.5	
C18:1ω9c	0.62		35.16	0–2.9	
C19:0*cyc*				0.6–1.8	
C19:1*cyc*		1.37	2.41		

*Strains: (1) Strain BFH1 (data from this study); (2), *Methylocaldum* sp. O-12 (Eshinimaev et al., 2004); (3) *Methylothermus thermalis* MYHT^T^ (Tsubota et al., 2005); (4) *Methylococcus capsulatus* (data from Bowman et al., 1993); (5) *Methylomagnum ishizawai* RD11D-Pr^T^ (Khalifa et al., 2015). Values are given as a percentage of total fatty acids.*

*Significance of bold values: to compare major fatty acids among methane oxidizers.*

### Analysis of Functional Genes and Phylogenetic Characterization

**FIGURE 3:**
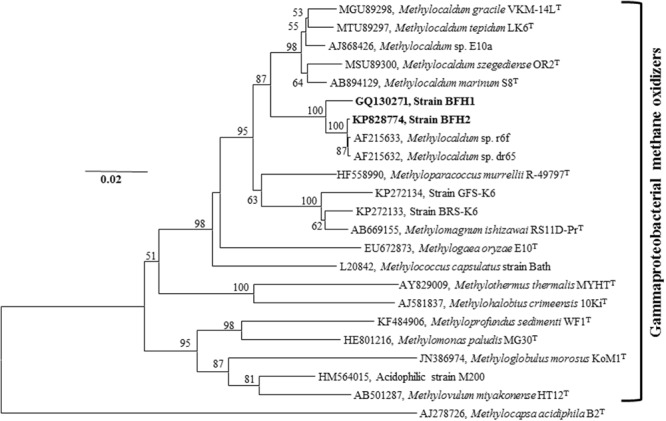
**16S rRNA gene-based Neighbor-Joining tree showing the phylogenetic placement of strains BFH1 and BFH2 (indicated in bold) among described gammaproteobacterial MOB.** The tree was constructed using MEGA6 software package. Bootstrap values are based on 1000 resamplings. Bar, 0.02 substitutions per nucleotide position. The type II alphaproteobacterial methanotroph *Methylocapsa acidiphila* (AJ278726) was used as an outgroup. GenBank accession numbers are given in front of the respective isolates name. The evolutionary distances were computed using the Kimura 2-parameter method and there were a total of 1200 positions in the final dataset. Bootstrap values less than 50% are not shown.

Polymerase chain reaction products were obtained from the functional genes *pmoA, mxaF*, and *cbbL* for both BFH1 and BFH2 (**Table 1**), whereas PCR amplification of the *mmoX* gene gave negative results. The absence of *mmoX* was verified by Southern hybridization using *mmoX* probe, which gave no signal with genomic DNA of the two strains (Supplementary Table S2). Phylogenetic analysis of the nearly full-length (1409 bp of strain BFH1 and 1429 bp of strain BFH2) 16S rRNA sequences revealed that both strains belonged to the class *Gammaproteobacteria*, and showed 97.8% sequence identity to each other. The strains clustered with moderately thermophilic and thermotolerant methanotrophs in the genera *Methylocaldum, Methylococcus*, and the mesophilic genera *Methyloparacoccus* and *Methylogaea* belonging to the family *Methylococcaceae* (**Figure 3**). BLASTn search of 16S rRNA genes revealed that the strains showed 97–99% sequence identity to clones of uncultured bacteria from industrial sugarcane bagasse feedstock piles (GenBank Accession No. HM362597, HM262490, HM362577, HM362564, HM362458, HM362551, HM362534–36, HM352524, HM352510; Rattanachomsri et al., 2011). The closest isolated relatives were the thermophilic methanotrophs *Methylocaldum* sp. dr65 (identity 97.6–99.7%) and *Methylocaldum* sp. r6f (identity 97.6–99.7%), isolated from landfill cover soils, *Methylocaldum* sp. E10a (identity 94.9–95.3%; Knief and Dunfield, 2005), and *Methylocaldum* sp. 05J-I-7 (identity 95.6%; GenBank Accession No. EU275146) isolated from landfill upland soil. The strains BFH1 and BFH2, however, formed a separate branch from *Methylocaldum* spp., which was supported by a high bootstrap value. Furthermore, pairwise sequence analysis of 16S rRNA genes of BFH1 and BFH2 with the closest described relatives showed only 93.1–94.1% sequence identity to *Methylocaldum* species (*M. szegediense* OR2^T^, *M. gracile* VKM 14L^T^, *M. tepidum* LK6^T^, *Methylocaldum marinum* S8^T^), 92.0–92.7% to *Methyloparacoccus* spp., 90.2-92.4% to *M. capsulatus* strain Bath, 90–90.8% to *Methylogaea oryzae* E10^T^, 87.9–88.4% to *M. thermalis* MYTH^T^ and 88.0–88.2% to acid-tolerant type I methanotrophic strain M200 (Supplementary Table S3). This indicates that our strains most probably represent a new genus of type I gammaproteobacterial methanotrophs. All 16S rRNA phylogenetic trees, Neighbor-Joining tree (**Figure 3**), Maximum-Likelihood tree (Supplementary Figure S2), and Minimum-Evolution tree (Supplementary Figure S4), exhibited the same topologies for strain BFH1 and BFH2, indicating that they were not members of other described methanotrophic genera in the families *Methylothermaceae* or *Methylococcaceae*.

**FIGURE 4:**
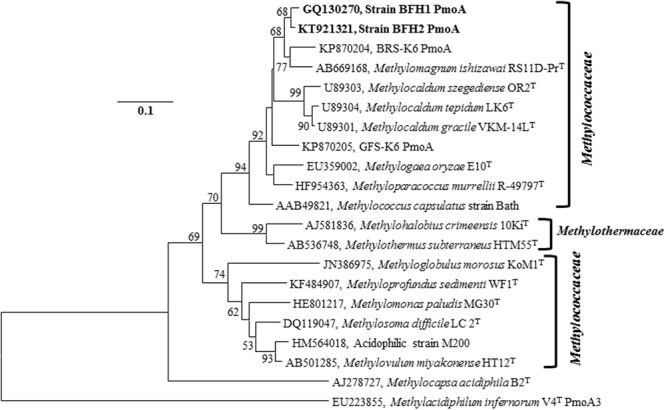
**PmoA Neighbor-Joining tree (based on predicted protein sequences) of the strains BFH1 and BFH2 related to other Gammaproteobacterial methane oxidizing bacteria.** GenBank accession numbers are given in front of the respective isolates name. Bootstrap values (1000 replicates) less than 50% are not shown. Bar, 0.1 substitutions per nucleotide position. The evolutionary distances were computed using the JTT matrix-based method and there were a total of 140 positions in the final dataset. The type II Alphaproteobacterial methanotroph *M. acidiphila* (AJ278727) and a thermoacidophilic verrucomicrobial methanotroph, *Methylacidiphilum infernorum* V4 PmoA3 (EU223855), were used as an outgroup. Evolutionary analyses were conducted in MEGA6.

BLASTp search of the PmoA amino acid sequence of the strain BFH1, showed 94% identity to uncultured methanotrophic clones from planted rice-field soil (GenBank Accession No. CA098534, AGO14814, and AFL65442), activated sludge (GenBank Accession No. BAG12175), and rice wetland (CBI68832). The closest cultured relatives of BFH1 and BFH2, based on PmoA sequences, were *Methylocaldum* spp. (similarity 94.9–96.8%), *Methyloparacoccus* spp. (94.9%), *M. capsulatus* strain Bath (93.7%), *M. oryzae* E10^T^ (93.1%), *M. thermalis* MYTH^T^ (86.7%) and the acid-tolerant type I methanotrophic strain M200 (87.3%; Supplementary Table S3). Furthermore, partial PmoA based trees, Neighbor-Joining tree (**Figure 4**), Maximum-Likelihood (Supplementary Figure S3), and Minimum-Evolution (Supplementary Figure S5), showed similar topology. It suggested that the isolates were well-separated from other taxonomically described representatives of type I methanotrophs.

BLASTp search of amino acid sequences of MxaF protein from strains BFH1 and BFH2, showed highest identity to *Methylocaldum* sp. 5FB and *Methylocaldum* sp. E10a (96–96.6%), *M. szegediense* (94%), *M. capsulatus* (93.4%), *Methyloparacoccus murrellii* strains R-49797^T^ and OS501^T^ (92.2–92.6%), *Methylomarinum vadi* IT-4^T^ and *Methylovulum miyakonense* HT12^T^ (88.5%) and *Methyloglobus morosus* Kom1^T^ (87.8%; Supplementary Table S4). CbbL sequences of strains BFH1 and BFH2 showed closest relationship to the methanotrophic bacteria *M. capsulatus* strain Bath (GenBank Accession No. AF447860) and *M. szegediense* (GenBank Accession No. WP_026609010) with 98.1 and 95.0% amino acid sequence identity, respectively.

## Discussion

Through cultivation efforts, two novel moderately thermophilic and acid-tolerant methane oxidizers, designated BFH1 and BFH2, were isolated from a methane-rich slightly acidic tropical topsoil habitat. The isolates were strictly aerobic and showed methylotrophic growth with methane and methanol as sole carbon and energy source. They showed optimal growth on LMA medium, which was 10 times more diluted than AMS medium and supplemented with NH_4_Cl.

Phylogenetic analysis of the 16S rRNA gene of the isolates revealed that they may represent a new clade within the family *Methylococcaceae* of the class *Gammaproteobacteria*, and this phylogenetic inference was supported by the physiological properties and chemotaxonomic analyses (**Tables 1** and **2**). The strain BFH1 differed significantly from characterized *Methylocaldum* spp. in major PLFA compositions, genomic DNA mol% G+C content (>4% difference), pH growth range (4.2–7.5 versus 6–8), and growth on methanol. The isolates are moderately thermophilic, not thermotolerant or true thermophilic, as they cannot grow above 65°C. Recently, a moderately thermoacidophilic methanotroph of the family *Methylothermaceae, Methylothermus subterraneus* HTM55^T^, was validly described and this bacterium is able to grow at temperature up to 65°C and at pH 5.2–7.5 (Hirayama et al., 2011). Three moderately acidophilic strains *M. paludis* MG30, M200 and M5, were the first isolates of type I methanotrophs that were recovered from an acidic wetland and *Sphagnum* peat bog, respectively (Kip et al., 2011; Danilova et al., 2013). These strains are psychrotolerant or mesophilic methanotrophs (temperature range 4–30°C), and able to grow between pH 3.8 and 7.3. Strain M200 is most closely related to the genus *Methylovulum* and probably represent a novel genus, whereas strains M5 and *M. paludis* MG30 are affiliated with the genus *Methylomonas*. However, these strains have been isolated from temperate environments. A number of type I methanotrophs of the family *Methylococcaceae* (genera: *Methylogaea* and *Methylomagnum*; strains: BRS-K6 and GFS-K6) from different tropical habitats (rice paddy fields and methane seep pond sediments) have been described. They are mesophilic (growth temperature range 8–37°C), neutrophilic (pH range 5–9) and non-thermotolerant (Geymonat et al., 2011; Islam et al., 2015; Khalifa et al., 2015). However, isolates BFH1 and BFH2 showed typical moderately thermophilic features (temperature range 30–60°C), and they were growing at pH values between 4.2 and 7, which indicates that they are most probably acid-tolerant or slightly acidophilic methanotrophs. This may reflect the isolates’ adaptation to the *in situ* pH and temperature of the tropical habitat they were derived from. Furthermore, pairwise distance analysis of the 16S rRNA gene showed a relatively high sequence identity (>97.6%) with the thermophilic methanotrophic isolates, *Methylocaldum* sp. dr65 and *Methylocaldum* sp. r6f, from landfill cover soils. The high sequence identity of these isolates to strains BFH1 and BFH2 suggests that they may have common physiology and metabolism properties.

Growth of the isolates was inhibited by acetylene, indicating that functional methane oxidation enzymes were present, whereas the naphthalene oxidation assay was negative, which verified the absence of soluble methane monooxygenase. These results are in accordance with those from other isolates of thermophilic or thermoacidophilic methanotrophs (Dedysh, 2009; Op den Camp et al., 2009). The genes *pmoA* and *mxaF* that encode key methane metabolism enzymes have been used as phylogenetic markers for both alphaproteobacterial and gammaproteobacterial methanotrophs. Detection of these genes in the isolates suggests that they produce these key methane oxidation enzymes. In addition, analyses of *mmoX* and Southern hybridization results indicate that the isolates do not have genes encoding the soluble methane monooxygenase. This gene is generally found in mesophilic and thermotolerant MOB, but not in moderately thermophilic or thermophilic MOB.

The major fatty acid in strain BFH was C16:0. This fatty acid is a major component in gammaproteobacterial thermophilic and thermotolerant (genera: *Methylocaldum, Methylothermus, Methylococcus*) methanotrophs (22–65%), whereas mesophilic and psychrotolerant type I methanotrophs (genera: *Methylomonas, Methylobacter, Methylomicrobium, Methylosoma, Methylosphaera, Methylosarcina*) contain relatively low C16:0 concentrations (4–18%; Bowman et al., 1997; Rahalkar et al., 2007; Kip et al., 2011). Exceptions are the mesophilic genus *Methylovulum* (47%) of type I methanotrophs (Iguchi et al., 2011), and the mesophilic genus *Methylohalobius* (23%; Heyer et al., 2005). The unsaturated fatty acid, C16:1*ω*7*c* was also found in strain BFH1. This fatty acid was not reported in the thermotolerant and moderately thermophilic genus *Methylocaldum*, whereas the thermotolerant genus *Methylococcus* contained about 10–23%, and the thermophilic genus *Methylothermus* had even lesser amounts (2–3.5%) of this unsaturated fatty acid (**Table 2**). On the other hand and interestingly, high amounts of this fatty acid (12–60%) are usually found in cold-adapted type I methanotrophs (Rahalkar et al., 2007; Kip et al., 2011; Deutzmann et al., 2014). Strain BFH1 also contained relatively high amounts of C17:0*cyc* (cyclopropane) as compared to other thermotolerant and thermophilic MOB. This makes its fatty acid composition unique, which can be used as a diagnostic feature, differentiating it from other methanotrophs.

In the present work, isolation of novel aerobic methanotrophs from methane-rich tropical soil has been achieved. No thermoacidophilic or acid-tolerant moderately thermophilic MOB has been described previously in the order *Methylococcales*. To the best of our knowledge, strains BFH1 and BFH2 are the first acid-tolerant moderately thermophilic methanotrophs of the class *Gammaproteobacteria* to be isolated. Therefore, the description of these strains will increase our knowledge of this ecophysiological group. The present study suggests that methanotrophs such as strains BFH1 and BFH2 may play a vital ecological role in methane-rich moderately acidic tropical habitats. They are obligate methylotrophs because they can only grow on methane or methanol. Together with *Methylocaldum* sp. dr65 and *Methylocaldum* sp. r6f they may represent one or more new species in a novel genus in the family *Methylococcaceae*. The new clade is probably diverse and widespread, particularly in tropical topsoil habitats. Future whole genome sequencing of the isolates together with biochemical and physiological analyses are expected to afford important insights into their ecophysiology and adaptation. The isolates are possibly coupled to the biogeochemically related reactions, and may provide evidences for controlling methane emissions to the atmosphere from the tropical surface gas blowout areas as well as evolutionary significance. The 16S rRNA, *pmoA, mxaF*, and *cbbL* sequences may assist for further identification of related methanotrophs from various habitats and show how these microorganisms are widely distributed. However, this study may improve our understanding of the methane oxidation process in such methane-rich tropical ecosystems, and extend our knowledge mainly based on studies of methanotrophs from temperate environments, which for instance indicate that enzymes involved in methane oxidation have average optimum temperature at 25°C (Aronson et al., 2013).

## Author Contributions

TI, VT, ØL, LB, LØ, and N-KB designed the experiments, analyzed the data, and wrote the manuscript. TI collected samples and isolated pure cultures and performed experiments. LØ and N-KB contributed reagents/materials/TEM analysis. VT has revised the manuscript.

## Conflict of Interest Statement

The authors declare that the research was conducted in the absence of any commercial or financial relationships that could be construed as a potential conflict of interest.

## Footnotes

1 http://web.expasy.org/translate
