# Supplementary material for: Acid-Tolerant Moderately Thermophilic Methanotrophs of the Class Gammaproteobacteria Isolated From Tropical Topsoil with Methane Seeps
Source: Front Microbiol. 2016 Jun 15;7:851. doi: 10.3389/fmicb.2016.00851 (PMC4908921; doi:10.3389/fmicb.2016.00851)

**Supplementary information**

**Table S1.** Primers for amplification of functional genes.

| **Genes** | **Primer sequences (5’🡪3’)** | **Product**  **(bp)** | **Results** | **Annealing temp. (^o^C)** | **Ref.** |
| --- | --- | --- | --- | --- | --- |
| *pmoA* | A189f: GGNGACTGGGACTTCTGG Mb661: CCGGMGCAACGTCYTACC | 510 | + | 55 | 1 |
| *mxaF* | f1003: GCGGCACCAACTGGGGCTGGT f1561: GGGCAGCATGAAGGGCTCCC | 558 | + | 58 | 2 |
| *cbbL* | McBCBBL 195F: CTGCTGACCGACCTCGACTA  McBCBBL 706R: GTCACGTTGAGGTAGTGGCC | 500 | + | 58 | 3 |
| *mmoX* | mmoXf882: GGCTCCAAGTTCAAGGTCGAGC mmoXr1403: TGGCACTCGTAGCGCTCCGGCTCG  f92: GGCTGCAGAGCTTYAMCTGGA  r1430: CGCCTCCCTCRTACTGYTCGAG | 535  1335 | −  − | 55  55 | 4  5 |

References:

1. Costello, A. M., Lidstrom, M. E. 1999. Molecular characterization of functional and phylogenetic genes from natural populations of methanotrophs in lake sediments. Appl. Environ. Microbiol. 65(11), 5066-5074.

2. McDonald, I. R., Murrell, J. C. 1997.The particulate methane monooxygenase gene pmoA and its use as a functional gene probe for methanotrophs. FEMS microbiol. Lett. 156(2), 205-210.

3. Baxter, N. J., Hirt, R. P., Bodrossy, L., Kovacs, K. L., Embley, T. M., Prosser, J. I., Murrell, J. C. 2002.The ribulose-1,5-bisphosphate carboxylase/oxygenase gene cluster of *Methylococcus capsulatus* (Bath). Arch. Microbiol. 177(4), 279-289.

4. McDonald, I. R., Bodrossy, L., Chen, Y. ,Murrell, J. C. 2008. Molecular ecology techniques for the study of aerobic methanotrophs. Appl. Environ. Microbiol. 74(5),1305-1315.

5. Islam, T., Jensen, S., Reigstad, L. J., Larsen, O., Birkeland, N. K. 2008. Methane oxidation at 55 degrees C and pH 2 by a thermoacidophilic bacterium belonging to the *Verrucomicrobia* phylum. Proc. Natl. Acad. Sci. USA. 105(1), 300-304.

**Table S2**. Results of Southern blot hybridization with radioactively labeled *pmoA* and *mmoX* probes.

| **Strains** | ***pmoA*** | ***mmoX*** | **Ref.** |
| --- | --- | --- | --- |
| Strains BFH1 and BFH2 | + | − | This study |
| *Methylococcus capsulatus* strain Bath | + | + | 1,2 |
| *Methylacidiphilum kamchatkense* strain Kam1 | − | − | 3 |

References:

1. Bodrossy, L., Kovács, K. L., McDonald, I. R., Murrell, J. C. 1999. A novel thermophilic methane-oxidising γ-Proteobacterium. FEMS Microbiol. Lett.170(2), 335-341.

2. Baxter, N. J., Hirt, R. P., Bodrossy, L., Kovacs, K. L., Embley, T. M., Prosser, J. I., Murrell, J. C. 2002.The ribulose-1,5-bisphosphate carboxylase/oxygenase gene cluster of *Methylococcus capsulatus* (Bath). Arch. Microbiol. 177(4), 279-289.

3. Islam, T., Jensen, S., Reigstad, L. J., Larsen, O., Birkeland, N. K. 2008. Methane oxidation at 55 degrees C and pH 2 by a thermoacidophilic bacterium belonging to the *Verrucomicrobia* phylum. Proc. Natl. Acad. Sci. USA. 105(1), 300-304.

**Table S3.** Pairwise sequence alignment analysis of 16S rRNA gene sequences and PmoA protein sequences shows similarity between the Thermoacid-tolerant strains BFH1 and BFH2, and other related methane oxidizing bacteria Ref. The European Molecular Biology Open Software Suite. Emboss Tools For Sequence Analysis. Available online: http://www.ebi.ac.uk/Tools/emboss/ (accessed on 25 August 2015). Identity of PmoA protein sequences shows in the parentheses. Values are given as a percentage.

| **Strains** | **BFH1**  **16S rRNA** | **BFH2**  **16S rRNA** | **BFH1**  **PmoA** | **BFH2**  **PmoA** |
| --- | --- | --- | --- | --- |
| **BFH1** | 100 |  | 100 |  |
| **BFH2** | 97.8 | 100 | 98.7 (98.1) | 100 |
| *Methylocaldum* sp. dr.65 | 97.6 | 99.7 | nr. | nr. |
| *Methylocaldum* sp. r6f | 97.6 | 99.7 | nr. | nr. |
| *Methylocaldum* sp. E10a | 95.3 | 94.9 | nr. | nr. |
| *Methylocaldum szegediense* OR2^T^ | 94.2 | 94.1 | 95.0 (90.6) | 91.7 (87.0) |
| *Methylocaldum gracile* VKM 14L^T^ | 93.7 | 93.7 | 96.8 (91.1) | 94.1 (88.2) |
| *Methylocaldum tepidum* LK6^T^ | 93.5 | 93.0 | 94.9 (89.9) | 91.7 (86.4) |
| *Methylocaldum marinum* S8^T^ | 93.3 | 93.1 | 96.8 (92.4) | 94.1 (89.3) |
| *Methylococcus capsulatus* Bath | 92.4 | 90.2 | 93.7 (87.3) | 92.3 (87.0) |
| *Methyloparacoccus murrellii* OS501 | 92.2 | 92.6 | 94.9 (91.8) | 93.5 (90.5) |
| *Methyloparacoccus murrellii* R-49797^T^ | 92.0 | 92.7 | 94.9 (91.8) | 94.9 (91.8) |
| ^a^GFS-K6 | 91.6 | 92.5 | 96.6 (92.6) | 94.7 (90.7) |
| *Methylomagnum ishizawai* RD11D-Pr^T^ | 91.5 | 92.2 | 96.8 (93.0) | 96.3 (93.3) |
| *Methylogaea oryzae* E10^T^ | 90.1 | 90.8 | 93.1 (88.3) | 92.6 (87.8) |
| *Methylohalobius crimeensis* 10Ki^T^ | 87.7 | 87.9 | 88.4 (78.1) | 87.1 (77.3) |
| *Methylothermus thermalis* MYTH^T^ | 88.4 | 87.9 | 86.7 (80.0) | 86.3 (80.4) |
| Acid-tolerant strain M200 | 88.0 | 88.2 | 87.3 (75.9) | 86.4 (75.1) |
| *Methylomonas paludis* MG30^T^ | 87.6 | 88.0 | 87.3 (76.6) | 85.2 (75.1) |

^a^A mesophilic gammaproteobacterial methane oxidizer of the family *Methylococcaceae* isolated from terrestrial methane seep pond sediments (Islam et al., 2015).

Reference:

Islam, T., Larsen, Ø., Torsvik, V., Øvreås, L., Panosyan, H., Murrell, J. C., Birkeland, N.-K., Bodrossy, L. 2015. Novel Methanotrophs of the Family *Methylococcaceae* from Different Geographical Regions and Habitats. Microorganisms 3(3), 484-499.

**Table S4.** Pairwise MxaF protein sequences similarity comparisons between BFH1 and other related MOB. Identity of MxaF protein sequences shows in the parentheses. Values are given in percentage. Ref. The European Molecular Biology Open Software Suite. Emboss Tools For Sequence Analysis. Available online: http://www.ebi.ac.uk/Tools/emboss/ (accessed on 10 September 2015).

| **Strains** | **BFH1 MxaF** | **BFH2 MxaF** |
| --- | --- | --- |
| **GQ130269, Strain BFH1** | 100 |  |
| **KT921322, Strain BFH2** | 99.4 (97.7) | 100 |
| AJ868416, *Methylocaldum* sp. E10a | 99.4 (96.0) | 98.9 (93.8) |
| AE017282, *Methylococcus capsulatus* Bath | 97.7 (93.4) | 97.2 (93.9) |
| HF954364, *Methyloparacoccus murrellii* R-49797^T^ | 98.3 (92.2) | 97.7 (92.0) |
| AJ868415, *Methylocaldum* sp. 5FB | 97.8 (96.6) | 97.2 (95.5) |
| DQ002935, *Methylocaldum szegediense* O-12 | 95.6 (94.6) | 96.2 (94.4) |
| U85503, *Methylocaldum tepidum* LK6 | 97.6 (95.9) | 97.1 (94.7) |
| DQ002936, *Methylocaldum szegediense* H-11 | 95.4 (94.0) | 96.0 (94.0) |
| AB453967, *Methylomarinum vadi* IT-4^T^ | 94.0 (88.5) | 94.4 (88.3) |
| AB501290, *Methylovulum miyakonense* HT12^T^ | 92.9 (88.5) | 93.3 (88.3) |
| HE801218, *Methylomonas paludis* MG30^T^ | 92.4 (87.5) | 92.2 (87.2) |

**Figure S1.** Growth of strain BFH1 at pH ranging from 3.5 to 8.0. The specific growth rate (μ h^-1^) at a given pH value was measured by assessing the cell numbers using phase contrast microscope at the beginning of the experiment and after 10 days incubation.

**Figure S2.** The evolutionary history was inferred by using the Maximum Likelihood method based on the Kimura 2-parameter model**,** using MEGA6 software package, of the 16S rRNA gene sequences of strains BFH1 and BFH2 related to other Gammaproteobacterial methane oxidizing bacteria. There were a total of 1200 positions in the final dataset. Genbank accession numbers are given in front of the respective isolates name. Bootstrap values (1000 replicates) less than 50% are not shown. Bar, 0.05 substitutions per nucleotide position. *Methylocapsa acidiphila* (AJ278726), an alphaproteobacterial type II methanotroph, was used as an outgroup.


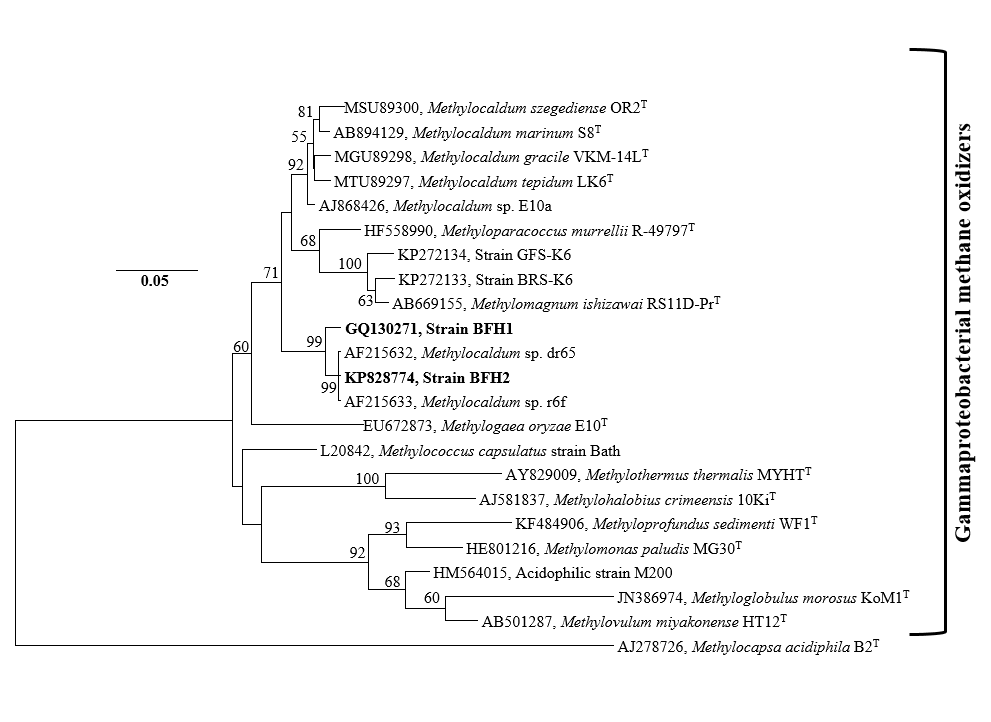


**Figure S3.** PmoA maximum-likelihood tree (based on predicted protein sequences) of the strains BFH1 and BFH2 related to other Gammaproteobacterial methane oxidizing bacteria. Genbank accession numbers are given in front of the respective isolates name. Bootstrap values (1000 replicates) less than 50% are not shown. Bar, 0.1 substitutions per nucleotide position. The evolutionary distances were computed using the JTT matrix-based method and there were a total of 140 positions in the final dataset. The type II Alphaproteobacterial methanotroph *Methylocapsa acidiphila* (AJ278727) and a thermoacidophilic verrucomicrobial methanotroph, *Methylacidiphilum infernorum* V4 PmoA3 (EU223855), were used as an outgroup. Evolutionary analyses were conducted in MEGA6.


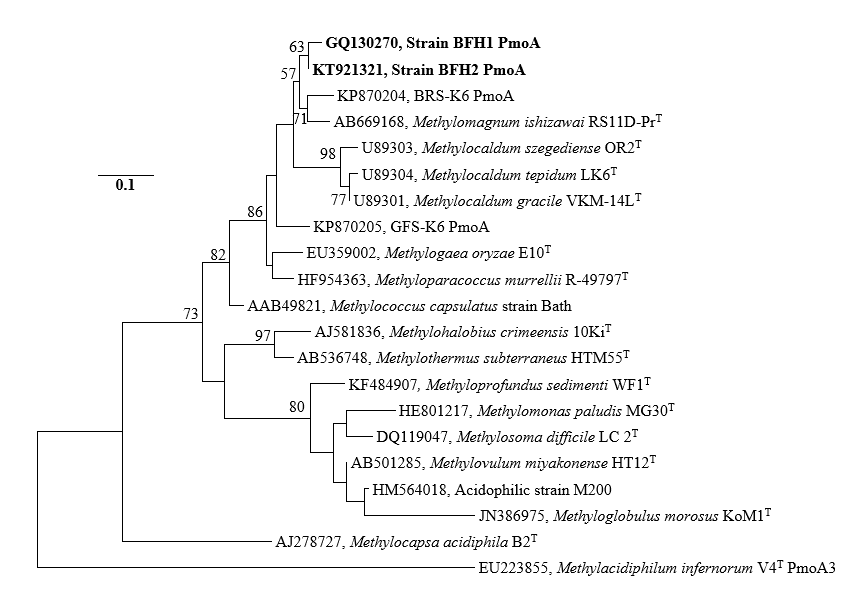


**Figure S4.** Minimum-evolution phylogenetic tree based on 16S rRNA gene sequences illustrating the relationships between strains BFH1, BFH2 and other related gammaproteobacterial methanotrophs. The evolutionary distances were computed using the Kimura 2-parameter method and evolutionary analyses were conducted in MEGA6. There were a total of 1200 positions in the final dataset. Genbank accession numbers are given in front of the respective isolates name. Bootstrap values (1000 replicates) less than 50% are not shown. Bar, 0.02 substitutions per nucleotide position. An alphaproteobacterial type II methanotroph, *Methylocapsa acidiphila* (AJ278726), was used as an outgroup.


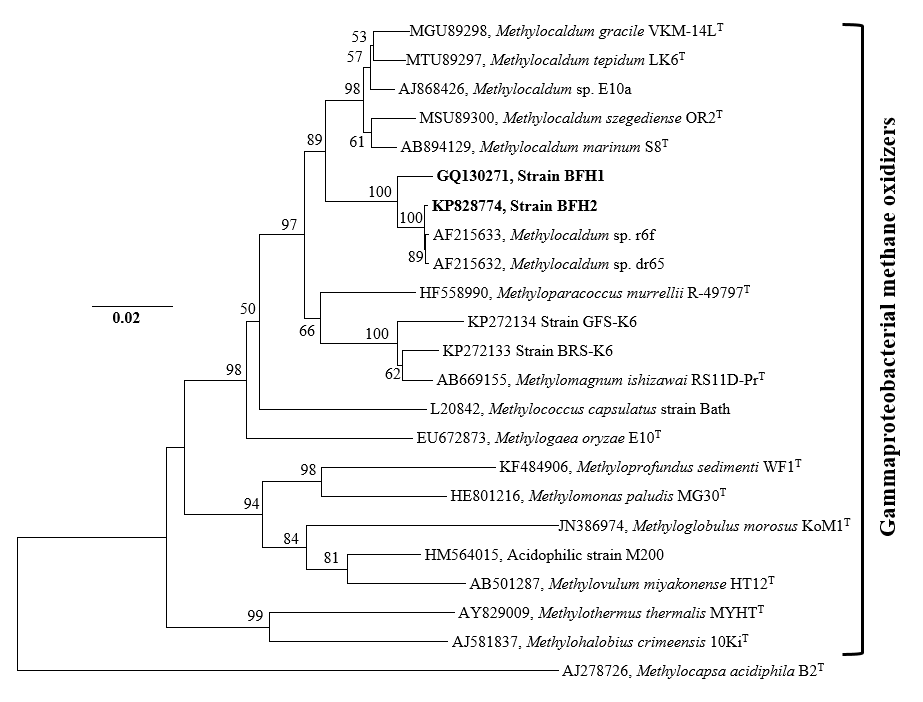


**Figure S5.** PmoA minimum-evolution tree (based on predicted protein sequences) of the strains BFH1 and BFH2 related to other Gammaproteobacterial methane oxidizing bacteria. Genbank accession numbers are given in front of the respective isolates name. Bootstrap values (1000 replicates) less than 50% are not shown. Bar, 0.1 substitutions per nucleotide position. The evolutionary distances were computed using the JTT matrix-based method and there were a total of 140 positions in the final dataset. The type II Alphaproteobacterial methanotroph *Methylocapsa acidiphila* (AJ278727) and a thermoacidophilic verrucomicrobial methanotroph, *Methylacidiphilum infernorum* V4 PmoA3 (EU223855), were used as an outgroup. Evolutionary analyses were conducted in MEGA6.


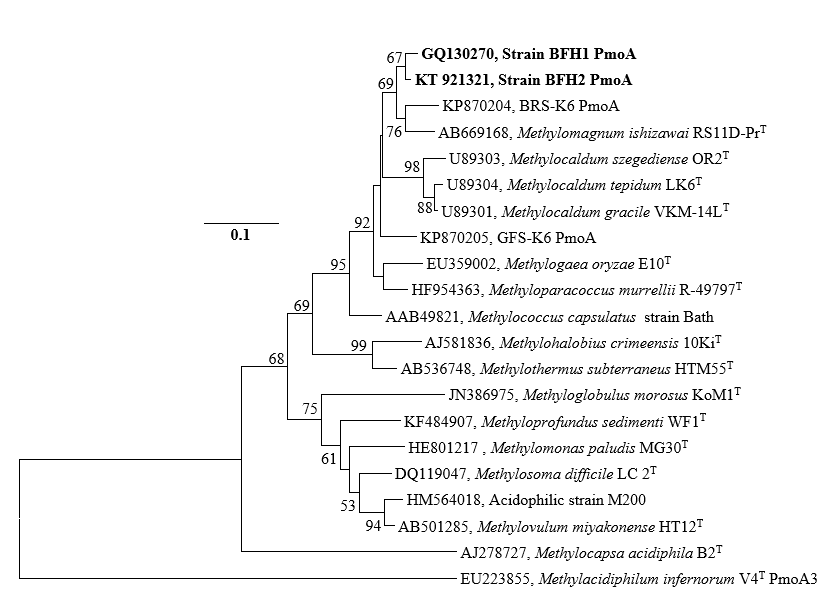

Supplement: Supplementary file 1 [file Table_1.DOCX]
